# Primary caregivers’ perceptions of challenges in dental and orthodontic care for children with Down syndrome

**DOI:** 10.1016/j.identj.2025.104016

**Published:** 2025-11-11

**Authors:** Elisabete Domingos Rebotim, Maria Björk, Henrik Berlin, Luc Marks, Malin Stensson

**Affiliations:** a The CHILD Research Group, School of Health and Welfare, Jönköping University, Jönköping, Sweden; b Centre of Odontology and Oral Health Sciences, School of Health and Welfare, Jönköping University, Jönköping, Sweden; c Department of Nursing, School of Health and Welfare, Jönköping University, Jönköping, Sweden; d Department of Paediatric Dentistry, Faculty of Odontology, Malmö University, Malmö, Sweden; e Faculty of Medicine & Health Sciences, University of Antwerp, Antwerp, Belgium; f Special Care in Dentistry, Department of Cranio-Maxillofacial Surgery, Antwerp University Hospital, Antwerp, Belgium

**Keywords:** Children, Down syndrome, Primary caregivers, Cooperation, Dental care

## Abstract

**Introduction and aims:**

Children with Down syndrome (DS) face increased oral health risks and barriers to dental care. Understanding caregivers’ perceptions about their child’s dental care is key to better outcomes. This study aimed to investigate primary caregivers’ perceptions of the dental and orthodontic care of children with DS, and the association with cooperation in encounters involving the child and dental professionals.

**Methods:**

A cross-sectional study was conducted using an online questionnaire completed by 77 primary caregivers, mostly mothers, of children with DS aged 6 to 17 years. Descriptive statistics and logistic regression were used to analyse the data.

**Results:**

Children with DS often needed support with toothbrushing, but were frequently reported as cooperative during home care and dental visits. Many children used alternative communication methods and had difficulty being understood. About half of the caregivers discussed their child’s needs with dental professionals in a premeeting. Fewer than half of the professionals were reported as communicating effectively with the children. Children who were perceived by their primary caregivers as cooperative during toothbrushing at home were significantly more likely to be cooperative during dental visits (*P* < .05). Additionally, being referred for an orthodontic assessment was statistically associated with higher levels of cooperation during dental appointments, according to the primary caregivers (*P* < .05).

**Conclusion:**

Despite communication challenges, many children showed cooperation during home care and dental visits. Perceived cooperation during toothbrushing at home and being referred for an orthodontic assessment were associated with higher cooperation during dental appointments for children with DS. Enhancing dental professionals’ communication skills and using personalised care approaches may improve dental outcomes and experiences for children with DS.

**Clinical relevance:**

These findings underscore the importance of tailored dental care approaches and improved communication techniques to foster active participation and better dental outcomes for children with DS.

## Introduction

Down syndrome (DS) is one of the most common congenital conditions, often accompanied by hearing and visual impairments, congenital heart anomalies and varying degrees of intellectual disability.^1^ In addition to these challenges, individuals with DS have increased susceptibility to poor oral health and oral diseases, particularly periodontal disease^2^ and malocclusion.^3^, ^4^, ^5^ This increased susceptibility to oral diseases arises from a combination of genetic, immunological,^6^ anatomical,^7^ and behavioural factors.^8^ For example, children with DS often have motor and musculoskeletal alterations. These alterations along with intellectual disability may affect their ability to effectively brush their teeth, exacerbating the risk of poor oral health.^9^ Malocclusion is highly prevalent in children with DS.^3^, ^4^, ^5^^,^^7^ This condition, along with other common features in DS, such as muscle hypotonia, can significantly affect oral functions such as chewing, breathing, and speech^4^^,^^10^ and decrease quality of life.^4^ When a child with DS is diagnosed with a malocclusion, an orthodontic assessment should be conducted to evaluate both the objective and subjective needs for treatment. Subjective needs refer to whether and how the malocclusion affects the child’s oral functions and the extent to which it impacts their overall well-being. The orthodontic assessment should be performed regardless of the underlying diagnosis, level of cooperation, or behavioural challenges.^3^^,^^5^ This is in line with the Swedish guidelines for the health and dental care of children with DS.^11^ Although malocclusion is a common condition among children with DS, research remains limited regarding the orthodontic care currently being provided to this population.

Children with DS are at increased risk of not receiving adequate dental care.^12^ The factors affecting dental care for these children can be child-related or environmental. Examples of environmental factors in this context may involve dental care organisations, professionals, and the child’s primary caregivers.^13^^,^^14^ Previous research has identified several environmental barriers as significant challenges to accessing health and dental care for children with DS. A common challenge is that dental professionals may lack the knowledge, training, or confidence needed to treat children with DS.^12^^,^^15^^,^^16^ Caregivers have also reported receiving insufficient guidance regarding the specific oral health needs of their child with DS.^17^ In broader healthcare settings, children with DS face barriers such as prolonged wait times for specialised care and poor coordination between healthcare providers.^18^ Studies further show that parents of children with DS tend to prefer dental professionals who have specialised knowledge of disabilities^10^^,^^19^^,^^20^ and who adopt a sympathetic, reassuring approach towards their child.^10^^,^^19^ A lack of these qualities can itself become a barrier to dental care. Likewise, child-related factors were reported as barriers in dental care for children with DS. Examples are behavioural and cooperation challenges, linked to intellectual impairments and communication difficulties.^5^^,^^8^^,^^12^^,^^21^

Cooperation, defined as intentional, mutual collaboration between two or more people^22^^,^^23^ is essential for effective dental care.^24^ In the context of dental care, cooperation arises from the interaction of multiple factors.^25^ Thus, for children with DS in the dental setting, cooperation should not be viewed solely as a behaviour of the child. It is rather a dynamic and interactive process involving both the child and the dental care provider. Establishing a collaborative relationship between the child and the dental professional is key to cooperation.^25^ This highlights the importance of the attitudes and communication skills of dental professionals in fostering cooperation and influencing the child’s involvement in the care process.^25^ In dental appointments, cooperation can be viewed as a context-specific expression of participation,^26^ particularly when participation is defined as involvement in a life situation.^13^ Cooperation can be considered as a specific dimension within the broader concept of participation. The International Classification of Functioning, Disability and Health (ICF) is a conceptual framework used to assess functioning and participation in individuals with health conditions or impairments,^13^ such as DS. It emphasises how the interaction between individual factors (body functions and structures) and environmental factors (physical, social, and attitudinal conditions) can support or hinder functioning. When such functioning occurs, it is considered an indicator of successful participation.^13^ According to the family of participation-related constructs, participation includes both attendance (‘being there’) and involvement (participating while attending).^14^ From this perspective, cooperation in dental care can reflect the child’s involvement in the dental care process. Participation is influenced by both child-related factors (such as the child’s abilities and motivation) and environmental factors.^13^^,^^14^ Cooperation, as a specific expression of participation, may also be shaped by these same factors. For instance, a child’s willingness to communicate, follow procedures, and engage in care-related tasks can reflect active involvement and participation. These behaviours serve as observable indicators of involvement and may be perceived by others as signs of the child’s cooperation. However, cooperation in its full complexity encompasses both the child and the dental professional, as well as the quality of their collaboration.^25^ An indicator of cooperation, such as following instructions and accepting care, reflects an active and functional interaction between the child and the dental professional. For cooperation to truly occur, the environment must actively nurture and support the child’s abilities to be involved in this process.^27^ Participation restrictions are environmental factors or barriers that hinder participation and involvement.^14^ For example, as reported in earlier research, children with DS may be excluded from accessing orthodontic treatment due to their diagnosis,^28^ limiting their opportunity to participate fully in care.

Yet little is known about the specific environmental and child-related factors that impact both appointment attendance and perceived cooperation during dental visits among children with DS. Identifying these factors is a key to promoting successful participation in oral healthcare, which is a fundamental right for all children.^29^ Therefore, this study aimed to investigate primary caregivers’ perceptions of the dental and orthodontic care of children with DS, and the association with cooperation in encounters involving the child and dental professionals. The following research questions were addressed:

(1) What child-related factors are associated with the child’s cooperation in dental care for children with DS according to their primary caregivers.
(2) What environmental factors are associated with the child’s cooperation in dental care for children with DS according to their primary caregivers.

## Material and methods

### Design and sample

This cross-sectional study used a study-specific questionnaire to collect data. A convenience sampling technique^30^ was used to recruit participants. The inclusion criterion was being a Swedish-speaking primary caregiver of a child with DS aged 6 to 17 years. The diagnosis of DS was based on self-report of the caregiver. Participants were recruited via social media and platforms targeting families of children with DS, habilitation centres, and public dental clinics in Sweden. Ethical approval was obtained (Dnr 2024-00982-01).

### Data collection

Administrators of specific social media (eg, Facebook, Instagram) accounts and groups affiliated with organizations targeting families of children with DS (eg, Swedish Association of DS) were contacted and invited to share information about the research study to their members. Upon agreement, brief information about the study and a web link were shared. Interested caregivers could then access detailed study information and complete the web questionnaire via the link provided (EsMaker Version 3.0, developed by Entergate AB).

Moreover, heads of departments at dental clinics and habilitation centres in four regions were also contacted and asked if posters with information about the study could be displayed in their waiting rooms. Once in the waiting room, interested caregivers could access further information about the study and the questionnaire through a provided QR code.

If the caregiver showed interest in participating, their informed consent was obtained digitally before accessing the questionnaire. Individuals who did not consent were automatically exited from the web questionnaire. Data collection lasted between May and October 2024 and was concluded after a sustained period without new responses. A final sample of 77 participants was selected.

### Instrument

A study-specific questionnaire was developed for this project to capture primary caregivers’ perceptions of dental and orthodontic care for children with DS. The development was informed by the ICF framework,^13^ the family of participation-related construct model,^14^ and the United Nations Conventions on the Rights of the Child and on the Rights of Persons with Disabilities,^29^^,^^31^ as well as by similar questionnaires used in France, Belgium, and Sweden.^10^^,^^19^^,^^32^^,^^33^

Item generation and refinement were conducted by three of the authors (MB, MS, ER) in collaboration with an expert panel (dentists, a dental hygienist, a nurse, and a disability expert) and a reference group (two primary caregivers of children with DS). The draft questionnaire was written in English, translated into Swedish, and then back-translated into English by three native Swedish speakers familiar with the field. Content validity and face validity were evaluated iteratively by both the expert panel and the reference group. Finally, one caregiver pilot tested the instrument, leading to minor adjustments.

The 40-question questionnaire covered six sections: (1) Demographic data about the child, (2) the child’s medical status, (3) the child’s activity and participation, (4) the role of environmental factors that influence participation, (5) accessibility and rights, (6) demographic data about the caregiver. For further information about the different sections and examples of questions, please see Table 1. The questionnaire included different response formats: a numeric scale (0-10) with anchors such as ‘*Not at all*’ to ‘*Completely*’, ‘*Not at all*’ to ‘*Very*’, and ‘*Very difficult*’ to ‘*Very easy*’ single and multiple options (eg, ‘*Yes, always*’, ‘*Yes, sometimes*’, ‘*No*’ and ‘*I don’t know*’), and open-ended questions (eg, ‘*If no, please explain why*’).

**Table 1:** Included sections in the questionnaire and examples of questions within each section.

Questions in sections 2-4 were developed by inspiration from different ICF domains and fPRC, section 5 from United Nations Convention.	
**Sections**	**Examples of questions**
**Section 1** Demographic data about the child	*Where does your child receive dental care?*
**Section 2** The child’s medical status (ICF domain body functions and structures and fPRC)	*Does your child have malocclusion, ie, the teeth and/or jaws are in the wrong position in relation to each other?*
**Section 3** The child’s activity and participation (ICF domain activity and participation and fPRC)	*Did your child cooperate with the dental staff at the last dental visit?*
**Section 4** The role of the environmental factors that affect participation (ICF domain environmental factors and fPRC)	*Has your child been able to practice the various elements that are included in a dental visit before the actual treatment is performed?*
**Section 5** Accessibility and rights (United Nations Convention on the rights of the child and rights of persons with disability)	*Do you think that your child is offered dental care in the same way as children without disabilities?*
**Section 6** Demographic data about the caregiver	*What is your level of education?*

### Analysis

Descriptive analyses, such as frequencies and medians, were used to characterise the participants. Scale variables were dichotomised into three categories (lower, medium, and higher values), and the 21 Swedish regions were grouped into three major regions (north, central, and south). Independent variables were tested for their association with cooperation in the dental appointment, the main outcome, using binary logistic regression. Variables were dichotomised, with continuous ones split into low (0-7/0-5) and high (8-10/6-10) values, and single/multioption responses grouped into two categories. Significant predictors (*P* ≤ .05) from univariate analysis were entered into multivariable models. The results were reported as odds ratios with 95% confidence intervals. Model fit was tested using Hosmer–Lemeshow and omnibus tests. Multicollinearity was checked using the Variance Inflation Factor and tolerance values. A Variance Inflation Factor >10 and tolerance <0.2 were considered indicators of multicollinearity.^34^ Analyses were done in Statistical Package for the Social Sciences (SPSS v28.0.1.0), with *P* < .05 indicating statistical significance. A descriptive, illustrative analysis was performed with the free-text responses. The themes derived from the statistical findings were used as a framework to organize the free-text responses. Quotations from primary caregivers with participant code numbers were used to illustrate variations observed in the quantitative findings.

## Results

### Sociodemographic characteristics of the study participants and their children

A total of 77 primary caregivers participated in the study. Most of the respondents had been born in Sweden (93.5%) and were mothers (90.8%). The participants’ mean age was 46.3 years (range 32-63 years). Regarding education, 81.3% held a college or university degree, and all of them (100%) reported oral health as very important. Regarding the children with DS, they had a mean age of 12.0 years (range 6-17 years), 51.9% were boys, and 48.1% were girls. Sociodemographic data about the children is provided in Table 2. Regarding the medical and dental background of the children, information is provided in Table 3.

**Table 2:** Sociodemographic data provided by children’s primary caregivers regarding their child with DS (*N* = 77).

Variables			*n* (%)
**Living status**	**Regions**	Northern regions	10 (13)
		Central regions	35 (45.5)
		Southern regions	32 (41.6)
	**Living arrangement**	At home with both parents	61 (79.2)
		Alternating residence with caregivers	8 (10.4)
		At home with one caregiver	7 (9.1)
		Short-term accommodation, alternating at home	1 (1.3)
**School**		Not in school yet	1 (1.3)
		Regular school	14 (18.2)
		Special school	61 (79.2)
		Finished school	1 (1.3)

**Table 3:** Medical and dental information provided by children’s primary caregivers regarding their child with DS (*N* = 77).

Variables			*n* (%)
**Regular contact with general healthcare services**			69 (89.6)
**Other diagnoses**		Autism spectrum	14 (17.9)
		ADHD/ADD	8 (10.3)
		Sleep apnoea (treatment with CPAP)	5 (6.4)
**Dental care**	**Delivery system**	Public	67 (87)
		Private	7 (9.1)
		Public and private	2 (2.6)
		I don’t know	1 (1.3)
	**Type of care**	General	19 (24.7)
		Specialist	31 (40.3)
		General and specialist	25 (32.5)
		I don’t know	2 (2.6)
**Use of sedation, nitrous oxide, or general anaesthesia for:**		Dental examinations	6 (7.9)
		Dental treatments	22 (28.9)
**Oral and orthodontic status**		Missing teeth	30 (39)
		Malocclusion	32 (42.1)

### Primary caregivers’ perceptions of their child’s dental care and child-related factors influencing the child’s cooperation in dental care

#### Factors related to the child’s behaviour

The primary caregivers reported that 79.2% of their children required significant assistance with toothbrushing at home. Among those who needed assistance, more than half (58.7%) were described as highly cooperative during toothbrushing, 25.3% were moderately cooperative, and 16.0% showed lower to no cooperation during toothbrushing. Regarding the last dental appointment, most of the primary caregivers (76.6%) experienced that their child was highly cooperative. Primary caregivers described the perceived cooperation of the child with dental professionals differently. One primary caregiver wrot*e: ‘He usually cooperates’* (Participant 13), whereas another primary caregiver shared: *‘Dentist visits are incredibly difficult. My son does not open his mouth’* (Participant 70).

The regression analyses showed that children who were perceived by their primary caregivers as cooperative during toothbrushing at home were more likely to be cooperative during dental visits (*P* < .05) (Table 4).

**Table 4:** Child-related factors predicting the cooperation of children with DS in dental care.

	Univariate			Multivariate	
Child-related factors	OR with 95% CI	*P* value	Included in final model	OR with 95% CI	*P* value
Observable cooperation of the child during toothbrushing at home	10.921 (3.484-34.228)	<.001	Yes	9.279 (1.705-50.482)	.010
Fear during dental appointment	0.232 (0.073-0.735)	.013	Yes	0.349 (0.44-2.739)	.316
Enjoyment when visiting the dental clinic	3.386 (1.226-9.346)	.019	Yes	1.511 (0.282-8.093)	.630
Communication skills of the child (being understood by others)	9.052 (1.929-42.472)	.005	Yes	0.825 (0.082-8.305)	.870
Aesthetics – How important the appearance of their teeth is to the child	1.263 (0.698-2.283)	.440	No	-	-

Results of the logistic regression. Hosmer–Lemeshow and Omnibus tests: The model was well adjusted. No multicollinearity was identified among independent variables.

#### Factors related to the child’s emotions

More than half of the participants (59.7%) reported that their child experienced little to no fear during dental visits, while 22.1% of children were moderately afraid, and 18.2% showed a high level of fear. Primary caregivers’ narratives showed the different levels of fear during dental visits. One primary caregiver wrote: ‘*Always goes well*’ (Participant 3) while another caregiver shared: *‘The child opened their mouth as best they could but was also scared and cried’* (Participant 43). In addition, 54.5% of primary caregivers reported that their child greatly enjoyed visiting the dental clinic, although 29.9% of children showed moderate enjoyment and 15.4% showed little to no enjoyment. One primary caregiver shared their positive feelings: *‘He sits in the chair, opens his mouth, and is happy and positive*’ (Participant 18), in contrast to another primary caregiver who wrote: *‘The child is too afraid to sit in the dentist’s chair’* (Participant 57). When asked about how important the appearance of their teeth was to the child, 65.7% of caregivers reported that, in their opinion, their child would consider dental aesthetics to be moderately to very important.

Factors related to the child’s emotions were not statistically associated with the perceived cooperation of the child during the dental visit (Table 4).

#### Factors related to the child’s communication

Regarding the child’s communication, 44.0% of the children communicated verbally, and 51.5% used sign language or images to support communication. Of the primary caregivers, 26.0% reported that their child was completely understood when communicating with others, whereas 42.9% reported that their child was moderately understood during communication, and 31.2% of children were minimally or not understood by others. Factors related to the child’s communication were not statistically associated with the perceived cooperation of the child during the dental visit (Table 4).

### Primary caregivers’ perceptions of their child’s dental care and environmental factors influencing the child’s cooperation in dental care

#### Factors related to the organisation

A total of 76.5% of primary caregivers perceived low or no difficulties when visiting the general dental care clinic with their child. The primary caregivers shared their experiences, with one expressing a positive experience by saying: *‘The dental care has exceeded expectations, as we have encountered great staff who took him seriously and included him’* (Participant 18) which contrasts with a negative experience from another primary caregiver who wrote: *‘Uncertain, what’s going to happen, who are we going to meet?’* (Participant 37). Regarding the specialist dental clinics, 70.7% perceived low to no difficulties when visiting these clinics. A primary caregiver shared: *‘I am very satisfied with the specialist dental care. They have been attentive to my son’s needs’* (Participant 30). However, another primary caregiver shared their negative experience: ‘*Waiting times, difficulties coordinating with other healthcare services. Forgetting the child’s perspective’* (Participant 71).

Most children (88.3%) were seen by the same dental professional at each visit, according to their primary caregivers’ reports. The participants also reported that 66.2% of the children underwent training before the actual treatment. One primary caregiver wrote: *‘Currently practising with the dental assistant. Last time, they were even able to tilt the chair back, which the child found uncomfortable during the previous visit’* (Participant 52). In contrast, 31.2% of the children did not have the opportunity for such training. Some caregivers (43.5%) perceived this training to be very important for their child. Additionally, one participant wrote: *‘We have been training him for the dentist since he was little because it was not possible to have a regular examination, which is why we now have a visit every month’* (Participant 46). Nearly all respondents (90.9%) perceived the duration of their child’s dental visits to be adequate. Factors related to the organisation were not statistically associated with the perceived cooperation of the child during the dental visit (Table 5).

**Table 5:** Environmental factors predicting cooperation of children with DS in dental care.

	Univariate		Multivariate		
Environmental factors	OR with 95% CI	*P* value	Included in final model	OR with 95% CI	*P* value
Dental professionals’ knowledge about communication	0.904 (0.487-1.678)	.748	No	–	–
Dental professionals’ knowledge about DS	0.873 (0.233-3.268)	.840	No	–	–
Difficulties going to a general dental care clinic	0.400 (0.100-1.599)	.195	No	–	–
Difficulties going to a specialist dental care clinic	0.202 (0.055-0.748)	.017	Yes	0.404 (0.071-2.308)	.308
Premeeting with the dental professional	0.373 (0.131-1.063)	.065	No	–	–
Preparatory training of the child before dental procedures	0.367 (0.109-1.239)	.106	No	–	–
Meeting the same professional	1.440 (0.314-6.607)	.639	No	–	–
Quality of dental care	1.086 (0.330-3.576)	.892	No	–	–
Primary caregivers’ satisfaction	1.524 (0.532-4.363)	.433	No	–	–
Equality in dental care	1.779 (0.647-4.892)	.265	No	–	–
Type of dental care	0.563 (0.163-1.940)	.363	No	–	–
Referred for an orthodontic assessment	11.870 (2.506-56.211)	.002	Yes	14.874 (1.927-114.806)	.010

Results of the logistic regression. Hosmer–Lemeshow and Omnibus tests: The model was well adjusted. No multicollinearity was identified among independent variables.

#### Factors related to dental professional’s knowledge and attitude

Half of the primary caregivers (50.6%) had a premeeting with dental professionals before the actual appointment to present their child’s needs, while 44.2% did not. Most caregivers (61.0%) perceived that dental professionals had sufficient knowledge about DS, 10.4% disagreed, and 28.6% were unsure. Nearly half of the primary caregivers (48.1%) believed that dental professionals communicated effectively with their child, followed by 29.9% who felt that communication was moderately effective and 22.1% who considered it insufficient. About dental professionals’ knowledge, primary caregivers shared their different opinions: ‘*I’m not sure if they have knowledge about Down syndrome, but they know what works and what we’ve developed together. We’ve previously used visual supports, among other things’* (Participant 46), contrasting with another primary caregiver who wrote: *‘Sign language should be mandatory for a dentist/dental assistant who is expected to communicate with someone who is unable to express themselves verbally’* (Participant 73). Primary caregivers’ narratives regarding dental professionals’ attitudes also showed opposite experiences and one participant shared: *‘They are absolutely fantastic, and he adored the dentist who played vampire with him’* (Participant 18) while another respondent shared a negative experience: ‘*I refused to go back there after the dental nurse called my child a “little mongoloid boy”’* (Participant 42). Factors related to the premeeting and the dental professionals’ knowledge and attitude were not statistically associated with the perceived cooperation of the child during the dental visit (Table 5).

#### Factors related to orthodontic care planification

An orthodontic assessment was recommended by the dentist for 36.4% of the children, while for half (57.1%), this option was not suggested, according to the primary caregivers. Only four children (5.2%) in the study were undergoing orthodontic treatment or had previously done so. One primary caregiver shared: *‘When she was little, we went to specialist dental care for the moulding of a mouth plate and follow-ups. Then we saw a regular dentist with support from the specialist’* (Participant 63). However, other participants wrote: ‘*They assume that my daughter won’t be able to handle having braces. According to them, this is all because the “Down syndrome” group doesn’t understand what it involves’* (Participant 19) and another participant shared: *‘My son will probably need to have some teeth extracted to make space (…) and it will not be followed by any further orthodontic treatment, as this is usually avoided for individuals with Down syndrome (as I understood it)’* (Participant 2). Additionally, being referred for an orthodontic assessment was statistically associated with higher levels of cooperation during dental appointments, according to the primary caregivers (*P* < .05) (Table 5).

### Factors related to the primary caregiver’s overall opinions of their child’s dental care

Almost all primary caregivers (85.7%) perceived that their child was treated well by the dental professionals. Also, 81.8% of the primary caregivers reported being satisfied overall with the dental care that their child received. Additionally, many primary caregivers (71.1%) perceived that their child received dental care in the same manner as children without disabilities. Regarding the caregivers’ overall satisfaction and opinion about dental care for their child with DS, they wrote about their wishes for change: ‘*It has generally worked quite well, but clearer communication about the dental health plan would have been helpful’* (Participant 69) and *‘Many children with Down syndrome never learn to chew food, like our child. I wish there were an action plan for this’* (Participant 9). Factors related to the primary caregiver’s overall opinion of their child’s dental care were not statistically associated with the perceived cooperation of the child during the dental visit (Table 5).

## Discussion

The results from this study showed that children with DS often required support with toothbrushing at home, and many were perceived to be cooperative during home care and dental visits. Furthermore, many children used alternative communication methods, and the primary caregivers perceived that some children had challenges in being understood. About half of the caregivers discussed with dental professionals their child’s needs in a premeeting. Only half of dental professionals were perceived as communicating effectively with these children. Furthermore, it was found that children who were perceived by their primary caregivers as cooperative during toothbrushing at home were significantly more likely to be cooperative during dental visits. Additionally, being referred for an orthodontic assessment was statistically associated with higher levels of cooperation during dental appointments, according to the primary caregivers.

The results revealed that most children with DS required adult support with their toothbrushing routines at home, a finding consistent with similar studies conducted in Sweden and Japan.^19^^,^^20^ Adult support is crucial because children with DS often face challenges with toothbrushing. These challenges can be caused by motor limitations and musculoskeletal issues that hinder their ability to effectively brush their teeth. This can lead to poor oral health and a higher risk of oral diseases.^9^ However, research also suggests that manual dexterity and hand function in children with DS can be improved through targeted training and support.^35^ Therefore, it may be important to implement supported and regular toothbrushing routines for children with DS to develop this ability over time. Furthermore, it was found in this study that children who were perceived by their primary caregivers as cooperative during toothbrushing at home were significantly more likely to be cooperative during dental visits. These findings suggest that enhancing the child’s cooperation in daily oral hygiene routines may facilitate cooperation during dental visits. Therefore, it is crucial to establish structured oral hygiene practices at an early age, as recommended by the Swedish guidelines.^11^ Also, the creation of familiarity with toothbrushing might improve the child’s cooperation during toothbrushing at a later age.^36^ Additionally, regular caregiver involvement in oral hygiene routines at home has been shown to predict better toothbrushing behaviour for children^37^ and to improve oral health outcomes for children with DS.^16^ This emphasises the importance of adult support during toothbrushing for these children. Dental professionals should educate the primary caregivers of children with DS on oral hygiene techniques and provide them with the necessary tools to better support their child’s oral health and oral needs. As evidence, research has shown that oral health training for caregivers positively impacts the oral health of individuals with disabilities.^38^ Strengthening collaboration and communication between dental professionals and primary caregivers of children with DS is vital to improving oral hygiene routines at home and dental care experiences for these children.^4^^,^^20^^,^^39^ In recent research, primary caregivers also expressed a need for more time to build a trusting relationships with their child’s healthcare providers.^18^

In the present study, the primary caregivers perceived that most children with DS did not have cooperation difficulties during the dental visit. Although sedation methods and general anaesthesia can facilitate cooperation during dental visits, they were not so often used by children in this study. This suggests that most children in this study could cooperate without the need for such methods. Another key technique to improve cooperation during dental appointments, especially for individuals with intellectual disabilities, is preparatory training,^39^, ^40^, ^41^ which is recommended in Sweden to support children with DS.^11^ In the present study, not all children had the opportunity to undergo this training, maybe because many children cooperated during their appointments. Also, the results showed that preparatory training of the child was not statistically associated with the perceived cooperation of the child during the dental visit. This suggests that while preparatory training is important, it may not be equally effective for all children. Other factors or techniques might play a more significant role in facilitating cooperation on the child level.

Children with DS often have communication difficulties^17^ that can affect cooperation in dental settings.^8^ This study found that many children with DS needed support tools to communicate, such as images or sign language, and frequently struggled to be understood, which can create barriers to dental care. Augmentative and alternative communication tools are known to support oral care for children with special needs.^42^ Moreover, effectively communicating with children in dental care was considered to facilitate the cooperation of the child in this context.^25^ However, in this study, only half of the dental professionals were perceived by the primary caregivers as able to communicate effectively with the child with DS. This ineffective communication between dental professionals and the child with DS can be a barrier to receiving dental care for children with DS. This study’s results showed that factors related to the child’s communication were not statistically associated with the perceived cooperation of the child during the dental visit. Nevertheless, prior research suggests that communication difficulties can contribute to noncooperation in children with DS.^21^ To improve cooperation and participation in dental care for children with disabilities like DS, dental professionals should have knowledge about supportive communication methods such as augmentative and alternative communication.^24^^,^^42^ A premeeting between dental professionals and the primary caregivers of children with DS can serve as an opportunity to share important information about each child’s specific communication methods. Despite this, many primary caregivers in the current study reported that they did not feel they had the opportunity to discuss their child’s needs with the dental professionals in a premeeting, before the dental visit. The reason for this lack of communication remains unclear and can be seen as a barrier to dental care for children with DS. Research indicates that primary caregivers of children with DS value communication skills and want dental professionals to use them when communicating with their child.^28^ A premeeting to inform dental professionals about the child’s communication needs enable the professionals to prepare and adapt their approach, ultimately enhancing communication and the child’s involvement and participation. The use of images or other communication tools in healthcare is fundamental to individualised care in paediatrics, providing a way for the child to more effectively communicate and actively participate in their care.^43^^,^^44^ Ensuring that children with DS are heard and understood respects their right to participate in healthcare decisions, regardless of their disability.^29^^,^^31^

So, in order to improve dental care for children with DS, dental professionals should provide individualised care that considers each child’s unique abilities, needs, and experiences,^39^ rather than focusing solely on their diagnosis.^45^ This perspective is even supported by research and by the World Health Organization as a means to improve health outcomes,^39^^,^^46^^,^^47^ and it aligns with the ICF framework. The ICF emphasises the role of external factors, such as the environment, on the overall functioning of the child, rather than only the body or the impairment.^13^ To implement this approach, dental professionals should gather information about the child’s daily routines, family dynamics, challenges during toothbrushing, sources of motivation, and types of support provided. Understanding these elements not only helps tailor care for each child but also creates opportunities to foster involvement and participation in dental visits. According to Imms et al,^14^ participation is influenced by the child’s preferences, motivation, and self-confidence. In the context of oral health, research shows that children are more likely to engage actively in care when they feel motivated and emotionally involved, and something as simple as letting them choose their own toothbrush can help foster these feelings.^48^^,^^49^

In this study, about half of the 77 children with DS were reported by their primary caregivers to have malocclusion, with a similar number referred for orthodontic assessment. This suggests that assessments were probably based on diagnosis. However, malocclusion may have been underreported, as the primary caregivers might be unaware of the condition, potentially leading to underestimated prevalence. This is consistent with the findings of Andersson et al,^3^ where all children with DS had some form of malocclusion as diagnosed by a dentist. Additionally, being referred for an orthodontic assessment was statistically associated with higher levels of cooperation during dental appointments, according to the primary caregivers. This association between perceived cooperation and orthodontic assessment may suggest that children with malocclusion who initially showed cooperation may have been more likely to receive an orthodontic assessment. Signs of early observable cooperation could have informally influenced the decision to suggest an assessment. However, the need for orthodontic assessment should be based primarily on the presence of malocclusion and its impact on the child’s quality of life and well-being,^3^ as also established in the Swedish guidelines for children with DS.^11^ In relation to orthodontic treatment, this study found that only a small number of children with DS had received or were currently undergoing such treatment, as reported by their primary caregivers. The low number may reflect the fact that some children were too young for treatment when the data were collected. Additionally, orthodontic treatment often requires a motivated and cooperative patient,^3^^,^^5^ which can be challenging in children with DS due to their intellectual disabilities.^50^ As a result, these children may be excluded from treatment even when malocclusion is present. This could help explain the current findings and aligns with Swedish parents’ perceptions of unequal access to orthodontic care for children with DS, as reported by Stensson et al. As previously discussed, to ensure that children with DS and malocclusion receive an orthodontic assessment, professionals must follow the Swedish national guidelines^11^ and focus on facilitating cooperation in the dental setting. This begins by seeing each child as an individual, not just a diagnosis, as a diagnosis alone does not determine behaviour or capability^45^ and assumptions about a child’s abilities based solely on their diagnosis should be avoided. Improving cooperation between the child and the dental professional may increase access to orthodontic care. However, while identifying factors that facilitate cooperation can help anticipate and maybe enhance cooperation, actual cooperation in practice also depends on the dynamic interplay between the child, the dental team, caregivers, and the environment. This understanding is essential for interpreting the findings of this study. This study has several limitations. The questionnaire was newly developed for this project and has not been previously applied to this population. To strengthen its credibility, we assessed content and face validity through expert review, caregiver input, and pilot testing with one caregiver. However, because the instrument was intentionally designed as a modular tool with heterogeneous item formats (numeric scales, multiple-choice, and open-ended questions), traditional psychometric analyses such as internal consistency and construct validity are not applicable. In addition, because the questionnaire was administered at a single time point, test–retest repeatability could not be evaluated in this study. Future research may explore the stability of responses over time in repeated administrations.

The sample size in this study was small and nonrepresentative of the broader population, with a predominance of highly educated, Swedish-born mothers. This demographic skew likely influenced the findings, as previous research by Chou et al^51^ has demonstrated that higher parental education is associated with more favourable child oral health behaviours. Consequently, certain groups, particularly fathers, individuals with lower education, and immigrant populations were underrepresented. Immigrant families may face barriers, including language difficulties and challenges in navigating health and dental services, especially in the absence of adequate support or translation services.^18^

Furthermore, fathers and mothers may differ in how their beliefs and behaviours impact children’s oral health outcomes.^51^ Additionally, Minervini et al,^52^ found that parental education level significantly influences their children’s oral health. This underrepresentation of these groups in this study may affect the generalizability of the findings. Participant recruitment targeted 100 individuals, consistent with similar studies.^10^^,^^19^ However, in the present study, the age range was more narrowly defined, including only children eligible for orthodontic consultation and treatment. The latter may have contributed to a lower parental response rate. Despite repeated dissemination of the questionnaire to encourage participation, the final response rate stabilized at 77, falling short of the intended target. Recruitment through digital channels may have excluded families with limited access to or familiarity with technology. The reliance on self-reported data from the primary caregivers introduces potential bias, as the participants may have been more informed or motivated than the general population. The main outcome, the child’s perceived cooperation at the last dental visit, may represent a one-time instance of cooperation. This only captures one moment in time and may not accurately reflect the child’s usual or overall pattern of cooperation. A child might have been more or less cooperative than usual on that day. So, while the measure gives useful information, it may not fully represent the child’s typical behaviour across multiple dental visits. Furthermore, the assessment of cooperation was based on the primary caregiver’s perception and own definition of this term, which introduces subjectivity into the outcome measure. As the present study focuses on the perception of parents and caregivers, it relied on self-reported data. No information was obtained from clinical records. Consequently, the children’s diagnoses and other conditions (eg, autism) were based solely on caregiver reports and were not clinically verified. Furthermore, data regarding the severity of these diagnoses were not collected, which limits the ability to assess how diagnostic complexity may have influenced the outcomes. Moreover, self-reported data restricts insight into the rationale behind dental professionals’ decisions regarding the child’s dental and orthodontic care. Another potential limitation is primary caregivers’ limited awareness of alternative orthodontic treatment options beyond conventional devices, which may have affected the accuracy and completeness of their responses. The small sample resulted in wide confidence intervals and imprecise estimates. While the associations remain statistically significant and clinically plausible, the magnitude of the effects should be interpreted with caution. Lastly, internal consistency reliability was not assessed since the questionnaire items were analysed as individual variables and not as composite scales. Similarly, test–retest reliability could not be evaluated as data collection occurred at a single time point.

Future research should include larger, more diverse, and representative samples. It should also incorporate the perceptions of children, dental professionals, and foreign-born primary caregivers, and further explore orthodontic care and cooperation strategies in this population.

## Conclusion

Despite perceived communication challenges in dental settings, many primary caregivers perceived that their child with DS cooperated with the dental professionals during dental visits. Furthermore, children who were perceived by their primary caregivers as cooperative during toothbrushing at home were significantly more likely to be cooperative during dental visits. Additionally, being referred for an orthodontic assessment was statistically associated with higher levels of cooperation during dental appointments, according to the primary caregivers. This underscores the important role of child-related but also environmental and contextual influences in shaping cooperation. The cooperation of children with DS in the dental setting is not solely driven by the child but is also significantly shaped by external factors such as primary caregivers, dental professionals, and the surrounding context. Although many children with DS relied on alternative communication methods and some had difficulty being understood, only half of the dental professionals were perceived as able to communicate effectively with them. This highlights an existing gap between communication needs and the perceived communication skills of dental professionals, which may have implications for dental care for children with DS. Furthermore, only about half of the primary caregivers had a premeeting with the dental professional to discuss their child’s specific needs before the appointment. This lack of opportunities for dental professionals and primary caregivers to collaborate and discuss the child’s individual needs, such as preferred methods of communication, may contribute to this gap. This highlights the importance of establishing a collaborative relationship between dental professionals and primary caregivers of children with DS. Also, dental professionals should adopt tailored dental care approaches and improve communication strategies when caring for children with DS in dental settings.

## Author contributions

The project was initiated by ER, LM, MB, and MS. ER collected data and took the main responsibility for analysing the data and drafting the manuscript. All authors (ER, MB, HB, LM, MS) have contributed to analysing the results and to drafting the manuscript.

## Declaration of generative AI and AI-assisted technologies in the writing process

During the preparation of this work, the author(s) used ChatGPT to improve the readability of the text. After using this tool/service, the author(s) reviewed and edited the content as needed and take(s) full responsibility for the content of the publication.

## Funding

This work was supported by the Research School at the School of Health and Welfare, Jönköping University, Jönköping, Sweden.

## Conflict of interest

The authors declare that they have no known competing financial interests or personal relationships that could have appeared to influence the work reported in this article.
